# Secretome Prediction of Two *M. tuberculosis* Clinical Isolates Reveals Their High Antigenic Density and Potential Drug Targets

**DOI:** 10.3389/fmicb.2017.00128

**Published:** 2017-02-07

**Authors:** Fernanda Cornejo-Granados, Zyanya L. Zatarain-Barrón, Vito A. Cantu-Robles, Alfredo Mendoza-Vargas, Camilo Molina-Romero, Filiberto Sánchez, Luis Del Pozo-Yauner, Rogelio Hernández-Pando, Adrián Ochoa-Leyva

**Affiliations:** ^1^Departamento de Microbiología Molecular, Instituto de Biotecnología, Universidad Nacional Autónoma de MéxicoCuernavaca, Mexico; ^2^Experimental Pathology Laboratory, Department of Pathology, National Institute of Medical Science and Nutrition “Salvador Zubirán”Mexico City, Mexico; ^3^Massive Sequencing Unit, National Institute of Genomic MedicineMexico City, Mexico; ^4^Thoracic Oncology Unit, National Institute of CancerMexico City, Mexico; ^5^Laboratorio de Estructura de Proteínas, National Institute of Genomic MedicineMexico City, Mexico

**Keywords:** secretome, *Mycobacterium tuberculosis*, antigenic density, drug targets, AAR, clinical isolates, bioinformatics, immunogenicity

## Abstract

The Excreted/Secreted (ES) proteins play important roles during *Mycobacterium tuberculosis* invasion, virulence, and survival inside the host and they are a major source of immunogenic proteins. However, the molecular complexity of the bacillus cell wall has made difficult the experimental isolation of the total bacterial ES proteins. Here, we reported the genomes of two Beijing genotype *M. tuberculosis* clinical isolates obtained from patients from Vietnam (isolate 46) and South Africa (isolate 48). We developed a bioinformatics pipeline to predict their secretomes and observed that ~12% of the genome-encoded proteins are ES, being PE, PE-PGRS, and PPE the most abundant protein domains. Additionally, the Gene Ontology, KEGG pathways and Enzyme Classes annotations supported the expected functions for the secretomes. The ~70% of an experimental secretome compiled from literature was contained in our predicted secretomes, while only the 34–41% of the experimental secretome was contained in the two previously reported secretomes for H37Rv. These results suggest that our bioinformatics pipeline is better to predict a more complete set of ES proteins in *M. tuberculosis* genomes. The predicted ES proteins showed a significant higher antigenic density measured by Abundance of Antigenic Regions (AAR) value than the non-ES proteins and also compared to random constructed secretomes. Additionally, we predicted the secretomes for H37Rv, H37Ra, and two *M. bovis* BCG genomes. The antigenic density for BGG and for isolates 46 and 48 was higher than the observed for H37Rv and H37Ra secretomes. In addition, two sets of immunogenic proteins previously reported in patients with tuberculosis also showed a high antigenic density. Interestingly, mice infected with isolate 46 showed a significant lower survival rate than the ones infected with isolate 48 and both survival rates were lower than the one previously reported for the H37Rv in the same murine model. Finally, after a druggability analysis of the secretomes, we found potential drug targets such as cytochrome P450, thiol peroxidase, the Ag85C, and Ribonucleoside Reductase in the secreted proteins that could be used as drug targets for novel treatments against Tuberculosis.

## Introduction

Worldwide, *Mycobacterium tuberculosis* (*M. tuberculosis*) remains a highly prevalent pathogen. According to the WHO, there were 10.4 million new cases and 1.4 million deaths in 2015 (WHO, 2016). Additionally, 3.3% of the new cases and 20% of the previously treated ones correspond to multidrug-resistant (MDR) infections (WHO, 2016). Although the use and development of rapid molecular diagnostic tests like Xpert MTB/Rif® and GeneXpert Omni® has expanded, the development of new drugs and vaccines is necessary. Moreover, with the wide genetic variation within *M. tuberculosis* strains and the impact that this variability has on the clinical outcome (López et al., 2003; Pérez-Martínez et al., 2008), there is a great need to understand the molecular mechanisms leading from strain genotype to the clinical phenotype. The strain H37Rv is the most studied *M. tuberculosis* strain, and it is an important model for laboratory studies. Another important *M. tuberculosis* family of strains is the “Beijing” genotype, a member of Lineage 2 (East-Asia), which has caused great concern because of their enhanced virulence, their highly transmissible phenotypes, and their increasing prevalence worldwide (López et al., 2003).

The complete set of Excreted/Secreted (ES) proteins, which is often referred as the cell secretome, is involved in critical biological processes, like mechanisms of adhesion, cell migration, and invasion, cell-to-cell communication, signal transduction and potential infective strategies in disease mechanisms (Tjalsma et al., 2004). As a facultative intracellular pathogen, *M. tuberculosis* relies on its ability to survive within the host through the secretion of virulent proteins with the capacity to modulate a variety of host cellular pathways (Smith, 2003; Målen et al., 2007; Chande et al., 2015; Vargas-Romero et al., 2016). ES proteins are an important source of immunogenic proteins due to their ability to be recognized by the host immune system. They are also considered T-cell antigens that promote protective immune responses against *M. tuberculosis* (Daugelat et al., 1992; Målen et al., 2007; Zheng et al., 2013). This has led to focusing most vaccine and drug development efforts to the identification of mycobacterial secreted proteins.

Several experimental attempts have been made to determine the secretome of *M. tuberculosis* strains, using “traditional” techniques such as 2-D gel electrophoresis or based on “omics” approaches like liquid chromatography coupled with different types of MS analysis (Målen et al., 2007). However, the molecular complexity of the pathogen cell envelope, composed by mycolic acids, peptidoglycan, acyl lipids, etc., complicate the experimental analysis of ES proteins (Zhou et al., 2015). To address this limitation, bioinformatics methods can be used for the systematized prediction of ES proteins from available sequenced genomes (Gomez et al., 2015). In this regard, two predicted *M. tuberculosis* secretomes were previously reported using bioinformatics approaches. In one study, the genome of H37Rv was screened to predict their encoded ES proteins using several secretion predictors, resulting in a secretome of 825 proteins (Vizcaíno et al., 2010). However, only one protein from each predictor was selected and experimentally confirmed as secreted (Vizcaíno et al., 2010). In a second study, the authors reported a database composed of 276 secreted proteins for the H37Rv genome using different bioinformatics algorithms (Roy et al., 2013) and they found that 46 from 57 experimentally confirmed secreted proteins were predicted in their secretome (Roy et al., 2013). However, neither of the two studies provided annotation analysis, biochemical pathway mapping, protein domain content or antigenic potential of their *M. tuberculosis* predicted secretomes. Also, the two reported secretomes could still contain transmembrane proteins because the algorithms used in their ES predictions do not analyze this type of proteins, plus, success in their ES prediction was only evaluated against few experimentally secreted proteins. In the present study, we sequenced and assembled two genomes of *M. tuberculosis* clinical isolates members of the Beijing genotype and the total encoded proteins were independently analyzed to predict the ES proteins for each genome. The predicted ES proteins were then annotated regarding sequence similarity to other known proteins, Kyoto Encyclopedia of Genes and Genomes (KEGG) pathways, gene ontologies (GO) and protein domains. Additionally, the antigenic density of the predicted secretomes was evaluated using the AAR value (Gomez et al., 2015). The secretomes for H37Rv, H37Ra and two BCG genomes were also predicted. Finally, a druggability analysis was also made for the predicted secretomes. We believe that our work could contribute to a better comprehension of the host-pathogen interactions in the *M. tuberculosis* infection.

## Methods

### Bacterial strains

We selected two *Mycobacterium tuberculosis* clinical isolates members of the Beijing genotype that we referred as isolates 46 and 48 in this manuscript. Prof D. van Soolingen at the National Institute of Public Health and the Environment (RIVM; Bilthoven, the Netherlands) kindly provided the isolate 46 that corresponds to the RIVM number 2002:1612 collected in Vietnam. This isolate is part of a tuberculosis isolates collection from a wide range of geographical origins. The isolate 48 corresponds to the previously described clinical strain code 1 reported in Tuberculosis patients attending primary health care clinics in the Western Cape Province of South Africa (Aguilar et al., 2010). This isolate was collected from the urban epidemiological field site in Cape Town during the period January 1993–December 2004 and kindly provided by Prof R. Warren from the Stellenbosh University (van der Spuy et al., 2009).

### Sequencing and assembly of *M. tuberculosis* clinical isolates

The bacterial genomic DNA (gDNA) of the 46 and 48 isolates was extracted from liquid cultures in logarithmic growth phase using the Quick-gDNA^TM^ MiniPrep kit after performing Zihel-Neelsen stains to assess the purity of *M. tuberculosis* cells. DNA libraries were constructed using the NebNext DNA Library protocol from Illumina (Cat. E6040S). Libraries were pair-end sequenced using Illumina GAIIx technology in the Unidad de Secuenciación Masiva from INMEGEN with a length of 72 bp per read and a sequencing depth of approximately 8 million paired reads per genome. The raw sequences were filtered using FastX-Toolkit and *de novo* assembled using Velvet (Zerbino and Birney, 2008). The resulting assemblies of each isolate are showed in Table S1. The final selected assemblies were analyzed with RAST (Aziz et al., 2008) to obtain the ORFs. We also extracted the ORFs from the H37Rv genome (GenBank: AL123456.3) using RAST to compare with the ones obtained for the clinical isolates.

### Prediction of ES proteins in *M. tuberculosis* genomes

**Figure 1:**
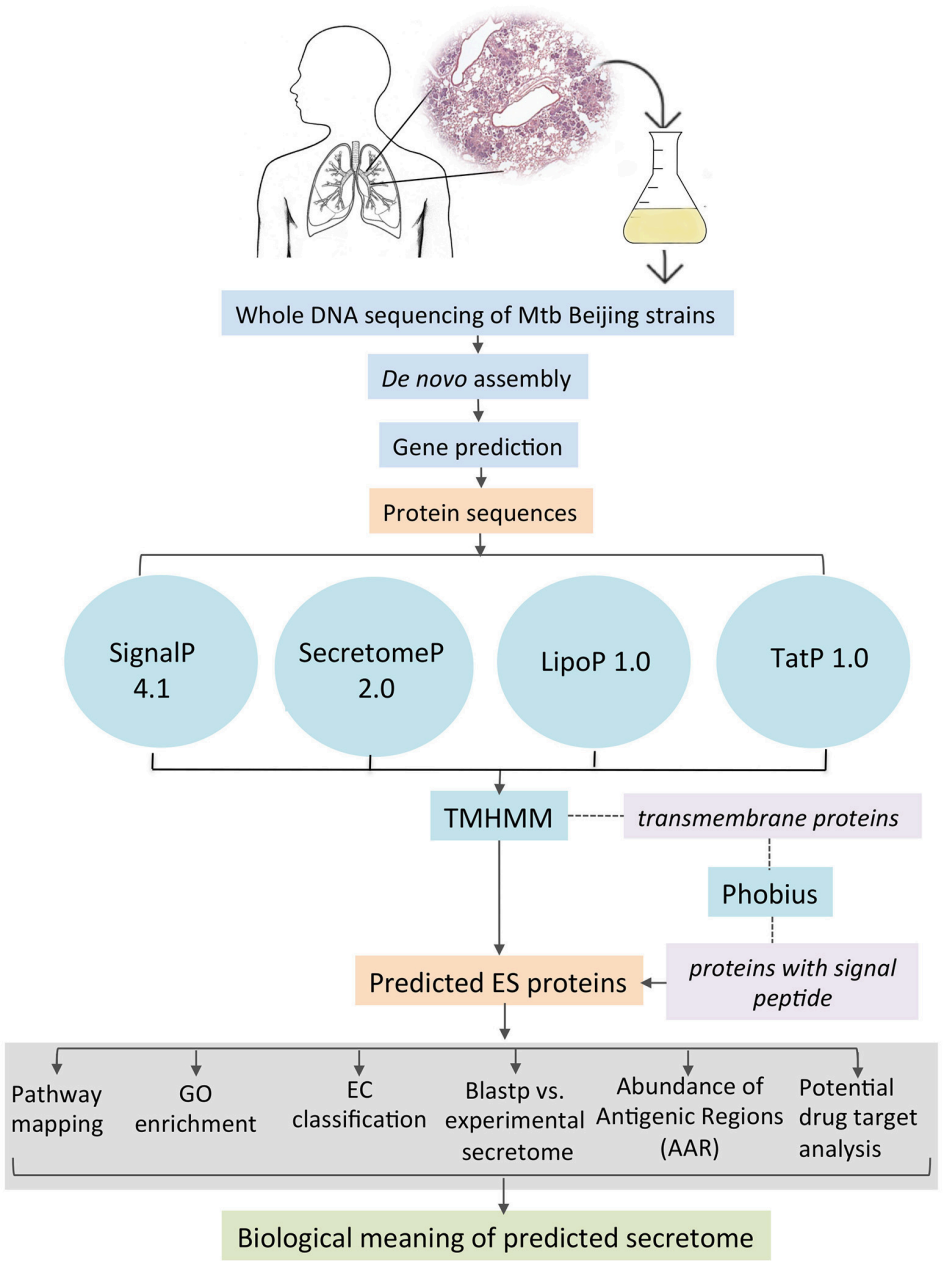
**Bioinformatics pipeline to identify and annotate the secretomes of *M. tuberculosis* genomes**.

All the coding gene sequences were analyzed independently for each genome by the different feature-based tools indicated in Figure 1. SignalP 4.1 (Bendtsen et al., 2004) was used to predict classically secreted proteins (Sec-dependent), setting the option for prokaryote organisms and the positional limit of 70 residues for truncation and the rest of the parameters were set as default. SecretomeP 2.0 (Bendtsen et al., 2005a) was used to predict the non-classical secreted proteins selecting the default options for Gram-positive bacteria and all the resulting proteins with an N-N score ≥ 0.5 were considered as positives. TatP 1.0 (Bendtsen et al., 2005b) was used to determine the proteins secreted via the Tat pathway applying the default parameters and the resulting proteins with a Tat motif were considered as positives. Additionally, we used LipoP 1.0 (Juncker et al., 2003) to predict lipoprotein motifs in the first 70 amino acids of each sequence, for this program, all settings were set to default, and all the resulting proteins with a “cytoplasmic” prediction were removed. Finally, all the proteins considered as positive from each of the predictors were merged together and the resulting list was scanned by TMHMM 2.0 (Krogh et al., 2001). This tool allows the identification, localization, and orientation of transmembrane helices and all the proteins predicted with 0 transmembrane motifs were assigned directly as part of the secretome. The rest of the proteins (with ≥1 transmembrane motifs) were further analyzed with Phobius (Käll et al., 2007) to identify possible α-helical conformations in the N-terminal region of the proteins that belongs to a signal sequence and that could be mistakenly classified as a transmembrane region. If any of the analyzed proteins was predicted to have a signal sequence it was added to the list of ES proteins. The secretome of the *M. tuberculosis* H37Ra GenBank CP000611 and two *M. bovis* BCG (BCG Danish GenBank NZ_CUWH01000001 and BCG Pasteur GenBank AM408590) strains were also predicted using the same bioinformatics pipeline. For comparison, the proteins that are neither ES and transmembrane was defined as “intracellular proteins.” Hence, the non-ES proteins consist of the transmembrane and the intracellular proteins.

### Annotation and comparative analysis of ES proteins

For identifying homolog proteins, ES proteins were analyzed using BLASTP against the non-redundant (nr) database using the Blast2GO (Conesa and Götz, 2008) with an E-value cut-off set at 1.0E^−3^ (Table S2). Both ES and non-ES proteins were functionally mapped to GO terms and annotated by setting the following parameters: *E*-value-hi-filter: 1.0E^−3^; Annotation cut-off: 55; GO weight: 5 and Hsp-Hit Coverage cut-off:0. The ES proteins were also associated with protein families through InterProScan (Zdobnov and Apweiler, 2001). Blast2GO was then used to identify the over or under represented GO terms in the ES proteins, by setting the term filter *p*-value to ≤ 0.05. Additionally, the KAAS (Moriya et al., 2007) was used for mapping ES proteins to KEEG pathways using the BBH (bi-directional best hit) method to assign the representative genes data set and the orthologs for prokaryotes (Table S3). We also classified the enzymes according to the six enzymes commission classes using Blast2GO (Figure S1).

### Construction of the experimental secretome

To validate the accuracy of our bioinformatics pipeline, we compared an experimental validated secretome (Figure S2) that we compiled from a literature search against our predicted secretomes. To construct the experimental secretome, we made a search at the NCBI database, and we retrieve all articles that experimentally reported excreted or secreted proteins for *M. tuberculosis*. After that, we ended up with 338 proteins that have been experimentally reported as secreted in different studies (Table S4). Then, we perform a BLASTP of the 338 proteins (*E*-value 1.0E^−3^) against our predicted secretomes to assess how many experimental ES proteins matched with the predicted secretomes. Only the secreted proteins reported as markers for serodiagnosis by Zhou et al. (2015), were also included in the experimental secretome. To do this, we analyzed the complete set of Zhou et al. (2015) with TMHMM and only the proteins without transmembrane regions were included in our experimental secretome.

### Calculation of the abundance of antigenic regions (AAR)

The AAR is a value used to normalize the number of antigenic regions by the sequence length (Gomez et al., 2015). This value was calculated as the ratio between the sequence length and the number of predicted antigenic regions for each protein and determines the number of amino acids that are needed to find one antigenic region within a protein sequence (Gomez et al., 2015). Hence, low AAR values mean that the protein has more antigenic regions (more antigenic density). We used the AAR value to evaluate the antigenic density of the different protein data sets. To this end, the number of antigenic regions for each protein sequences was obtained using BepiPred (Larsen et al., 2006) with the default settings (threshold 0.35) and normalized by sequence length (Gomez et al., 2015). The Mann-Whitney statistical test (*p* < 0.001) was used to establish if there is a significant difference between AAR values of protein data sets. To assess if the AAR observed in the predicted secretomes is significantly different to random constructed secretomes, we compared the AAR of the predicted secretomes to the AAR of 1000 protein datasets with 553, 519, and 548 randomly selected proteins from 46, 48, and H37Rv genomes, respectively. We then determined the AAR for each of the 1000 iterations and determined an empirical *p*-value by keeping track of the number of iterations equaled or exceeded the observed AAR for each corresponding secretome.

### Potential drug target analysis

We performed a BLASTP (E-value 1.0E^−3^) between the proteins of the 46, 48, and H37Rv secretomes to obtain the shared proteins in the three secretomes (core secretome). The resulting set of 449 shared proteins was further searched for sequence similarity against known drug targets available on the Drug Bank database (http://www.drugbank.ca/), setting the *E*-value to 1.0E^−3^ and the rest of the options to default. In Table S5 all the proteins that have similarity with a known drug target, as well as the drugs that can affect said target, are showed.

### Survival and drug resistance assays

The survival rate caused by the two clinical isolates was evaluated in 6- to 8-week-old male BALB/c mice as previously described (Hernandez-Pando et al., 1996). Briefly, two groups of 50 mice were each inoculated intratracheally with 2.5 × 10^5^ bacilli of each of the two clinical isolates in 100 μL Phosphate-Buffered Saline (PBS) and survival rate was recorded since day 1–day 90 post-infection. The clinical isolates were also evaluated for drug resistance (rifampicin, ethambutol, streptomycin and isoniazid) with the BD BACTECH™ MGIT™ 960 Mycobacteria Culture System following the manufacturer's recommendations.

### Nucleotide sequence accession numbers

This Whole Genome Shotgun project has been deposited at DDBJ/ENA/GenBank under the accessions MSLU00000000 and MSLV00000000. The versions described in this paper are MSLU01000000 and MSLV01000000.

## Results

### Genome sequencing and assembly

We sequenced the whole genome of the 46 and 48 *M. tuberculosis* clinical isolates, which were originated of patients from Vietnam and South Africa, respectively. Bacterial genomic DNA was extracted from liquid cultures and sequenced using Illumina GAIIx technology with a depth of approximately 8 million paired reads per genome. After genome assembly, we obtained 151 contigs for isolate 46 and 144 contigs for isolate 48 (Table S1) with approximately 71- and 72-fold genome coverage of a 4.3 Mb genome size, respectively. The Open Reading Frames (ORFs) were extracted for each genome, resulting in 4336 and 4310 proteins for isolate 46 and 48, respectively. Additionally, we also extracted the ORFs in the H37Rv genome to compare with the proteins of the clinical isolates.

### Prediction of *M. tuberculosis* secretomes

Proteins can be secreted through multiple secretory mechanisms. Thus, we utilized a combination of different bioinformatics tools based on Neuronal Network (NN), Hidden Markov Model (HMM), and Support Vector Machine (SVM) algorithms to predict the mycobacterial ES proteins encoded in our assembled genomes. To this end we utilized SignalP 4.1, SecretomeP 2.0, TatP 1.0, LipoP 1.0, followed by TMHMM 2.0 and Phobius (Figure 1). Such algorithms have a high performance in predicting signal peptides, protein subcellular localization, and transmembrane proteins. In addition, several of these algorithms have good performance to predict signal peptides in mycobacterial proteins (Restrepo-Montoya et al., 2009; Vizcaíno et al., 2010). SignalP and SecretomeP 2.0 were used to determine the classical and non-classical secreted proteins, respectively. Lipoproteins containing signal peptides were identified using LipoP 1.0 and proteins containing a twin-arginine signal peptide cleavage site were predicted using TatP 1.0. Then, the proteins predicted as secreted through the four algorithms were merged, yielding a set of 1956 different proteins for isolate 46, 1920 different proteins for isolate 48 and 1285 proteins for H37Rv. Next, each protein dataset was analyzed for the presence of transmembrane regions by TMHMM 2.0 algorithm. All the proteins containing transmembrane regions were removed, and the remaining proteins were considered the secretome for the genome 46, 48, and H37Rv. The proteins with transmembrane motifs that were removed from each isolate were re-analyzed with Phobius to identify proteins with a α-helical conformation in the amino section that could have been mistakenly classified as a transmembrane region (Figure 1). From Phobius analysis, only 1 protein for isolate 46 was predicted to have a signal peptide and therefore it was added to the secreted list of proteins. For isolate 48 and H37Rv no proteins were predicted to have a signal peptide by Phobius. Finally, the predicted secretome of isolates 46, 48, and H37Rv consisted of 553, 519, and 548 proteins, respectively, which represent ~12% of the total proteins encoded in their *M. tuberculosis* genomes.

### Annotation of *M. tuberculosis* secretomes

**Figure 2:**
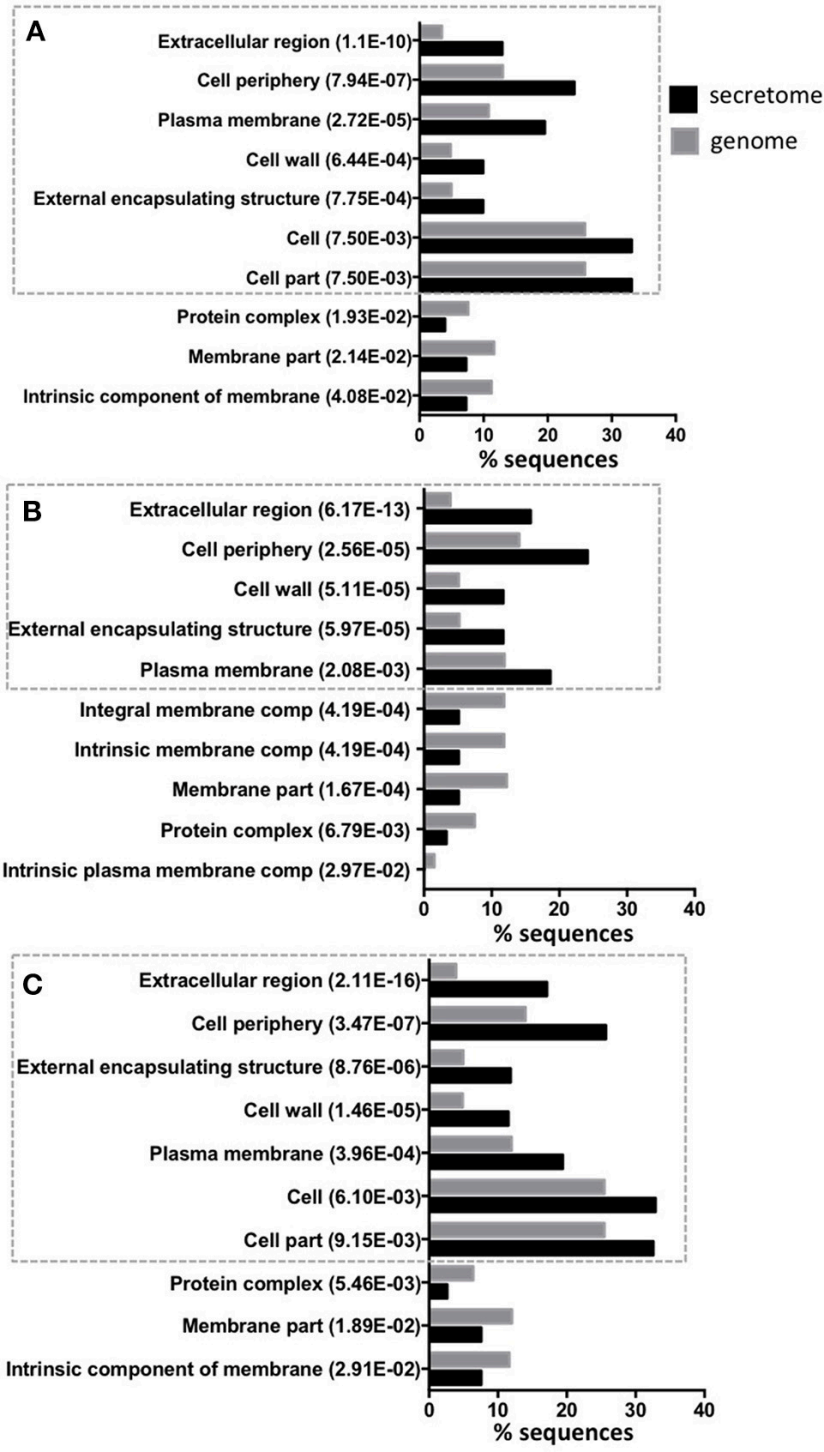
**Top 10 of significant over or under represented GO terms in predicted secretomes**. The figure shows for each significant GO term in Cellular Component category the amount (percentage) of sequences annotated with this term in secretomes and genomes. The Y-axis shows significantly overrepresented (inside the ticked lines) or underrepresented GO terms in secretome and the X-axis gives the percentage of sequences of each term. The *p*-value in parentheses, shows the statistical assessment of over/under representation of GO term in secretomes when compared to genomes. Isolate 46 **(A)**, isolate 48 **(B)**, and reference strain H37Rv **(C)**.

Of the predicted secretomes, 448 proteins (81.01%) of isolate 46, 484 proteins (93.26%) of isolate 48, and 502 proteins (91.6%) of H37Rv strain showed significant similarity (BLASTP matches) with proteins deposited in the non-redundant (nr) database. Furthermore, according with the BLASTP match, most of the ES proteins for the three strains were identified as members of the PE, PPE, and PGRS families (Table S2) which are known as important in the bacterial virulence mechanisms (Brennan and Delogu, 2002). ES proteins were then annotated for Biological Process, Molecular Function, and Cellular Components with GO terms using Blast2GO. This resulted in 316 (57.14%) proteins for isolate 46, 283 (54.53%) proteins for isolate 48 and 309 (56.39%) proteins for H37Rv that were annotated with 304, 392, and 115 different GO terms, respectively. We analyzed whether any GO term showed a statistically significant over or under representation in the secretome as compared to the expected GO term distributions for the whole genome of each strain. For the three strains a significant over/under representation was only observed in the Cellular Component category. As expected, the most over represented GO terms in the secretomes were extracellular region, cell periphery and external encapsulating structure (Figure 2), which are the typical cellular components reported for secreted proteins (Gomez et al., 2015). While, GO terms that are non-related to cellular components of secreted proteins such as membrane part, protein complex or intrinsic membrane component were under represented in the secretome (Figure 2). We also used KAAS for mapping the ES proteins to KEGG pathways. After that, a total of 132 (23.9%), 117 (22.5%), and 121 (22.08%) ES proteins of isolates 46, 48 and H37Rv secretomes were mapped to 74, 75, and 76 KEGG pathways, respectively. The two most frequently mapped KEGG pathways for each secretome were: ABC transporters and pyrimidine metabolism. Additionally, we found two proteins involved in beta-Lactam resistance pathway (Table S3). The Tuberculosis KEGG pathway groups all human and *M. tuberculosis* proteins that have been involved in host-pathogen interactions and as expected five proteins were mapped to this pathway: lipoprotein LpqH, lipoprotein LprG, phosphate transport system substrate-binding protein pstS, acid phosphatase SapM, and the 6 kDa early secretory antigenic target ESAT-6 (Table S3). The full pathway annotations are available in Table S3.

We also classified the enzymes of the ES and non-ES proteins according to the six Enzyme Commission (EC) Classes. The results showed an overrepresentation of oxidoreductases, isomerases, and hydrolases in the ES proteins of isolate 46 as compared to the same enzyme types for the non-ES proteins (Figure S1). For isolate 48, there is an overrepresentation of the same kinds of proteins besides ligases (Figure S1B) while in strain H37Rv there is an overrepresentation of hydrolases, isomerases, oxidoreductases and ligases (Figure S1C). The annotation of protein domains contained in the secretomes was conducted using InterProScan and resulted in 274 protein domains for isolate 46, 234 for isolate 48 and 253 protein domains for H37Rv. The most represented protein domains are shown in Table 1. For the three secretomes, the most represented protein domains were PPE family C-terminal, and PE-PGRS family N-terminal. Interestingly, these protein domains are involved in the *M. tuberculosis* pathogenicity (Fishbein et al., 2015).

**Table 1:** **Top 10 most represented protein domains in isolate 46, 48 and H37Rv secretomes**.

**InterPro code**	**InterPro description**	**Number of ES proteins (%)**
**ISOLATE 46**		
IPR022171	PPE family C-terminal	18 (3.25)
IPR012338	Beta-lactamase/transpeptidase-like	18 (3.25)
IPR000084	PE-PGRS family N-terminal	17 (3.07)
IPR016040	NAD(P)-binding domain	15 (2.71)
IPR027417	P-loop containing nucleoside triphosphate hydrolase	14 (2.53)
IPR029058	Alpha/Beta hydrolase fold	12 (2.17)
IPR012336	Thioredoxin-like fold	11 (1.99)
IPR029063	S-adenosyl-L-methionine-dependent methyltransferase	11 (1.99)
IPR000253	Forkhead-associated (FHA) domain	8 (1.45)
IPR017853	Glycoside hydrolase superfamily	7 (1.27)
**ISOLATE 48**		
IPR000084	PE-PGRS family N-terminal	29 (5.59)
IPR012338	Beta-lactamase/transpeptidase-like	20 (3.85)
IPR022171	PPE family C-terminal	20 (3.85)
IPR029058	Alpha/Beta hydrolase fold	18 (3.47)
IPR016040	NAD(P)-binding domain	15 (2.89)
IPR012336	Thioredoxin-like fold	14 (2.7)
IPR027417	P-loop containing nucleoside triphosphate hydrolase	14 (2.7)
IPR029063	S-adenosyl-L-methionine-dependent methyltransferase	9 (1.73)
IPR000253	Forkhead-associated (FHA) domain	8 (1.54)
IPR001763	Rhodanese-like domain	6 (1.16)
**REFERENCE STRAIN H37Rv**		
IPR000084	PE-PGRS family, N-terminal	68 (12.41)
IPR022171	PPE family, C-terminal	22 (4.01)
IPR016040	NAD(P)-binding domain	14 (2.55)
IPR029058	Alpha/Beta hydrolase fold	9 (1.64)
IPR027417	P-loop containing nucleoside triphosphate hydrolase	8 (1.46)
IPR012338	Beta-lactamase/transpeptidase-like	8 (1.46)
IPR012336	Thioredoxin-like fold	5 (0.91)
IPR007312	Phosphoesterase	5 (0.91)
IPR026954	PknH-like extracellular domain	4 (0.73)
IPR005490	L,D-transpeptidase catalytic domain	4 (0.73)

### The experimentally reported ES proteins confirm the accuracy of our predicted secretomes

To validate the accuracy of our bioinformatics pipeline to predict experimental secretomes, we compiled a protein dataset of 338 proteins (Table S4) experimentally reported as excreted/secreted in *M. tuberculosis* (see Methods) and determined how many proteins of this experimental secretome were also reported in our predicted secretomes. After that, we found that 227 (67.15%), 220 (65.09%), and 257 proteins (76.04%) of the experimental secretome were also contained in our predicted secretomes of isolates 46, 48, and H37Rv, respectively (Figure S2). These data indicates that ~70% of the experimental secreted proteins were also included in our predicted secretomes, showing that our bioinformatics method is quite accurate. To asses, if the same number of experimental secreted proteins could be found in a list of randomly selected proteins, we constructed 1000 random secretomes consisting of groups of 553, 519, and 548 randomly selected proteins from the 46, 48, and H37Rv genomes, respectively and matched each random secretome against the experimental one. After that, we found that the maximum percentage of shared proteins obtained by a random secretome was of 40%, which is lower than the percentage obtained with our predicted secretomes (~70%), indicating that our results are significantly different than random.

Additionally, we also matched the experimental secretome that we compiled from the literature search with the ones previously reported by Roy et al. (2013) and Vizcaíno et al. (2010). However, we found that only the 34.32 and 41.42% of the experimental secretome was shared with the secretome reported by Roy et al. (2013) and Vizcaíno et al. (2010), respectively (Figure S2). These results suggest that the bioinformatics pipeline we used is better to predict a complete set of excreted/secreted proteins in *M. tuberculosis* genomes.

### The AAR value reveals a high antigenic density in the predicted and experimental secretomes

**Figure 3:**
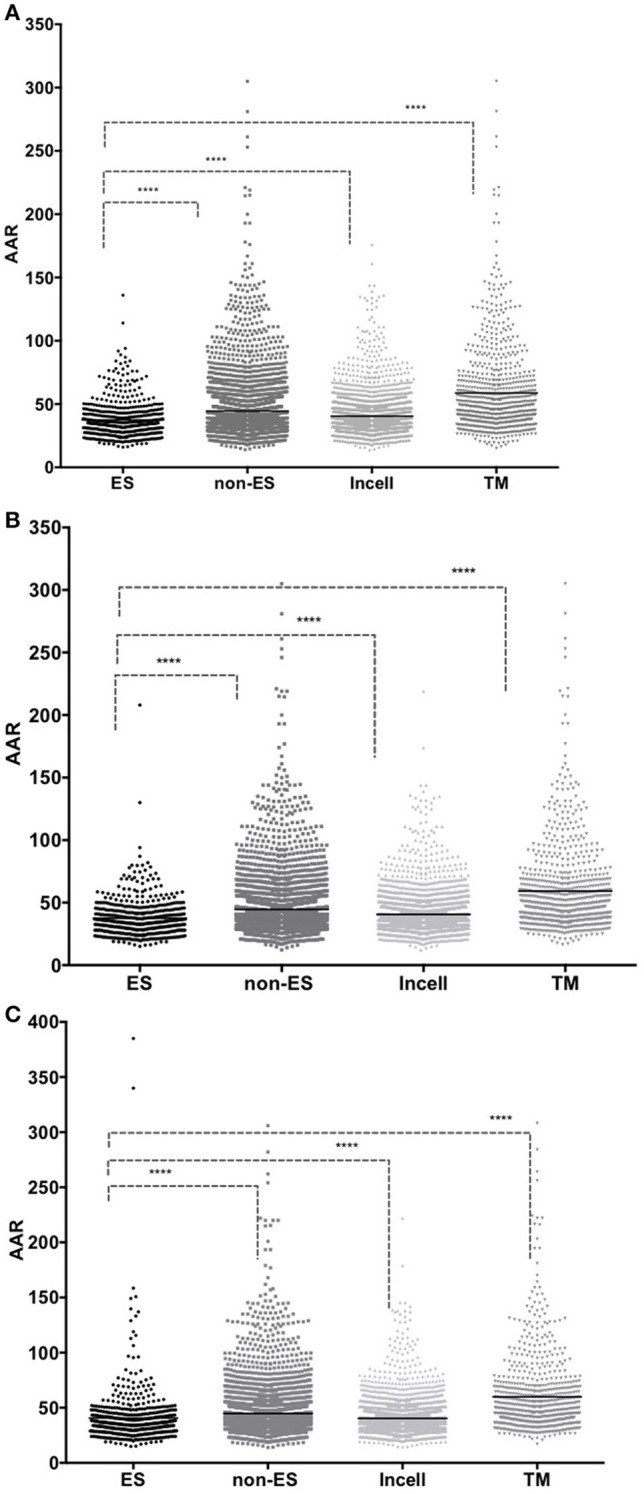
**Comparison between AAR values for Excreted/Secreted (ES), non Excreted/Secreted (non-ES) intracellular (Incell) and transmembrane (TM) proteins**. The number of antigenic regions was calculated using the BepiPred method. The X-axis shows the protein group analyzed and the Y-axis shows the AAR obtained for each protein in that dataset. Low AAR values means that a protein has more antigenic density. The AAR comparisons between the proteins of isolate 46 **(A)**; isolate 48 **(B)** and H37Rv **(C)** are illustrated. A Mann-Whitney test was performed to compare the AAR within each group with a confidence level of 99% (*p* ≤ 0.01), (^****^p < 0.001).

The AAR was used to calculate the antigenic density of the ES, intracellular, transmembrane and non-ES proteins of the isolates 46 and 48 and the H37Rv genomes. We found that the ES proteins have significantly more antigenic density (non-parametric Mann-Whitney test *p* ≤ 0.01) than the rest of the proteins encoded in the *M. tuberculosis* genomes (Figure 3 and Table 2). Interestingly, the secretomes of both clinical isolates had more antigenic density (AAR = ~37.5) than the H37Rv secretome (AAR = 40.6) (Table 2). However, no significant difference between each secretome after performing the Mann-Whitney test was observed. This result suggests that no strain seems to have a more antigenic secretome than the other even there was a tendency to more antigenic secretomes for the clinical isolates respect to H37Rv.

**Table 2:** **Abundance of Antigenic Regions (AAR) for *M. tuberculosis* predicted and experimental secretomes**.

	**ES Proteins**		**Non-ES proteins**	
***M. tuberculosis*** **strain**	**Number of proteins in the dataset**	**AAR average**	**Number of proteins in the dataset**	**AAR average**
Beijing isolate 46	553	37.52	3702	44.54
Beijing isolate 48	519	37.55	3743	44.56
H37Rv reference strain	548	40.63	3788	44.74
Experimental secretome	338	38.99	-	-

To validate the biological significance of the high antigenic density (lower AAR values) observed in our secretomes, we also calculated the antigenic density for the experimental secretome obtained from the literature search (Table 2). Interestingly, the antigenic density for the experimental secretome was similar to the one obtained for the predicted secretomes (Table 2). It has been reported that some parasite secretomes have more antigenic density that non-secreted or transmembrane proteins (Gomez et al., 2015; Wang et al., 2015). So, we also analyzed if the high antigenic density is exclusive of the *M. tuberculosis* secretomes or if any set of similar number of proteins could obtain the same AAR value. To this end, we selected 1000 groups of 553, 519, and 548 randomly selected proteins from the 46, 48, and H37Rv genomes, respectively, and calculated the AAR for each group (see Methods). After that, we found that all the predicted secretomes had more significantly antigenic density (*p* < 0.005) than the randomly constructed ones. Hence, the high antigenic density obtained for the predicted secretomes is exclusive of that combination of secreted proteins. In addition, to test whether the antigenic density of our predicted secretomes was similar to other *M. tuberculosis* secretomes, we applied our bioinformatics pipeline to obtain the secretome of the H37Ra, BCG Danish, and BCG Pasteur genomes and calculated their AAR values (Table 3). We selected the genome of H37Ra because it is an attenuated strain closely related to the virulent H37Rv strain. We also selected two substrains of the *M. bovis* Bacille Calmette-Guérin (BCG) strain because it is the bacteria used in the Tuberculosis vaccine. Interestingly, the antigenic density for the BCG secretomes was very similar to the ones obtained for our clinical isolates (Table 3). However, the AAR values of the clinical isolates still show a lower tendency to have more antigenic density, followed by BCG and H37Ra and H37Rv strains (Table 3).

**Table 3:** **Abundance of Antigenic Regions (AAR) for *M. tuberculosis* strains from different lineages**.

	**ES Proteins**	
***M. tuberculosis*** **strain**	**Number of proteins in the dataset**	**AAR average**
Beijing isolate 46	553	37.52
Beijing isolate 48	519	37.55
H37Rv reference strain	548	40.63
H37Ra	554	40.52
BCG Danish	526	38.99
BCG Pasteur	564	38.89

Taking the advantage of the fact that there is a lot of information about immunogenic proteins in Tuberculosis, we investigated if these immunogenic proteins have a high antigenic density. To this end, we selected two sets of proteins causing seropositive reactions in serum samples of Tuberculosis patients and determined their AAR values. The first set contains 57 secreted proteins resulted from a screening with 10 Tb patient and 3 healthy serum samples used as negative controls (Zhou et al., 2015). The second set contains 12 proteins characterized as serum biomarkers that can differentiate between both TB patients with active disease or recovered individuals; this second set was obtained from 189 patients (Deng et al., 2014). Interestingly, the AAR values were 38.5 for the former and 37.8 for the later protein dataset, suggesting that both sets of immunogenic proteins are composed of proteins with a high antigenic density.

### Predicted secretomes suggest novel drug targets

After a BLASTP (*E*-value 1.0E^−3^) comparison between the 46, 48, and H37Rv secretomes we found that 449 proteins were shared between the three secretomes. This set of proteins was named the “*M. tuberculosis* core secretome” and we compared their sequence similarity against known drug targets available on the DrugBank database to determine if some ES proteins could be used as potential drug targets in the host-pathogen interactions (see Methods). Of the 449 ES proteins, only 26 showed homology with 91 known drug targets (Table S5). Notably, of all possible drug targets, only a few have a known inhibitor activity.

### Survival and drug resistance assays

**Figure 4:**
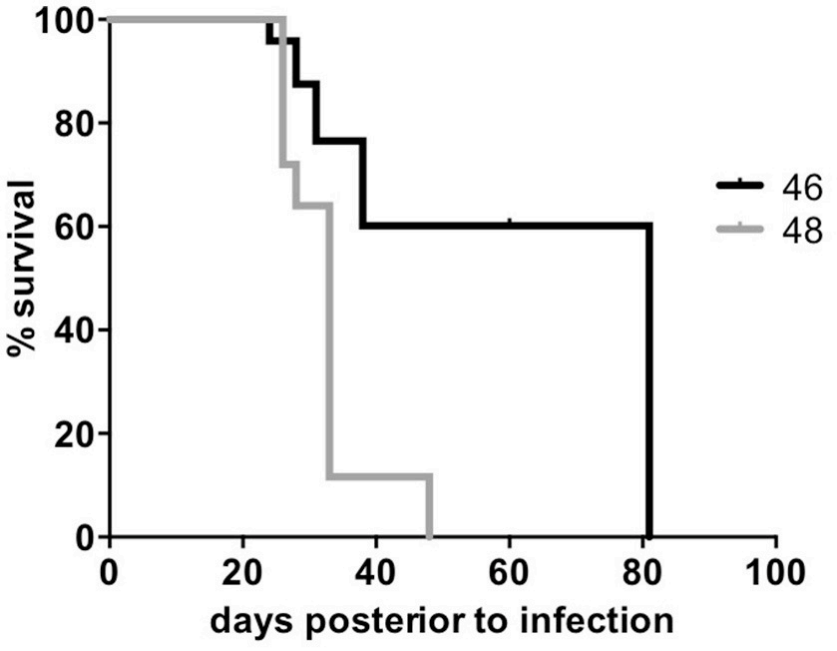
**Survival rate of Beijing clinical isolates**. The two *M. tuberculosis* Beijing clinical isolates were used to infect BALB/c mice and record the survival rate. The X-axis shows the days post-infection and the Y-axis shows the percent of survival. A Log-rank (Mantel-cox) test was performed to compare the survival percentages within each group.

We tested the survival rate of the 46 and 48 clinical isolates in a murine model (see Methods). Mice infected with isolate 46 showed the lowest survival rate, reaching 0% at day 48, while mice infected with isolate 48 survived until day 82 (Figure 4). After performing a log-rank (Mantel-Cox) test a significant difference was observed between the two survival rates with a p value ≤0.0001. Additionally, the two isolates also showed sensibility to rifampicin and ethambutol and resistance to streptomycin and isoniazid (see Methods).

## Discussion

To the best of our knowledge, this study contains the most comprehensive *in silico* and experimental collection of *M. tuberculosis* secretomes and it is the first one that takes into account the secretome analysis of clinical isolates. Our results showed that our bioinformatics pipeline is quite accurate to predict a complete set of the secreted/excreted proteins in *M. tuberculosis* genomes. In this regard, our data indicates that ~70% of the experimental secretome was also predicted as secreted using our bioinformatics approach, while only the 34.32 and 41.42 % of the experimental secretome was found in the secretomes reported by Roy et al. (2013) and Vizcaíno et al. (2010), respectively. Furthermore, the maximum coincidence of the experimental secretome against 1000 randomly constructed secretomes was of 45.3%, indicating that our predicted secretomes are also significantly different to the random.

The predicted ES proteins for the three *M. tuberculosis* genomes represented around ~12% of the total genome proteins. Interestingly, this value is twice the percentage reported for the secretomes of parasite organisms like tapeworms (Gomez et al., 2015; Wang et al., 2015). We suggest that this difference between the percentages of secreted proteins among parasites could be associated with the fact that parasites such as *T. solium* are extracellular pathogens while *M. tuberculosis* is an intracellular parasite that requires different invasion mechanisms with their host. The annotation of the secretomes showed an enrichment of the antigenic protein families such as the PPE and PE-PGRS (Table 1 and Table S2). It has been observed that these proteins may play a role in evasion of host immune responses, possibly via antigenic variation (Chaitra et al., 2008; Sampson, 2011; Akhter et al., 2012). As expected, several immunodominant antigens widely known for *M. tuberculosis* such as ESAT-6, Ag85C, CFP21, and CFP-10 (Silva et al., 2003; Wang et al., 2005) and several proteins of the Tuberculosis KEGG pathway (Table S3) were also present in our predicted secretomes. The overrepresentation of hydrolases and oxidoreductases in our predicted secretomes (Figure S1) is in agreement with the enrichment of this enzyme types reported in experimental *M. tuberculosis* secretomes (Målen et al., 2007). It is recognized that T-cells primarily mediate the immune response against an intracellular pathogen like *M. tuberculosis*. However, secreted and transmembrane proteins have also been identified to be targeted by B-cells in other intracellular bacteria like *Listeria* and *Chlamydia* (Grenningloh et al., 1997; Bannantine et al., 2000). In this regard, we chose BepiPred algorithm to analyze the antigenic density of the ES proteins, which predicts linear B-cell epitopes using Hidden Markov Models (Larsen et al., 2006). Interestingly, the predicted secretomes showed a significant higher antigenic density than the non-ES proteins (Figure 3). Additionally, a high antigenic density was also observed for the experimental secretome (Table 2). These results are in agreement with the high antigenic density reported for secretomes of 14 helminth species, including the human parasite *T. solium* (Gomez et al., 2015).

The antigenic density observed for the secretomes of the avirulent H37Ra and the virulent H37Rv strains was very similar, suggesting that the antigenic density is not associated with the virulence in these two strains. In fact, the avirulent phenotype of H37Ra is mainly associated to the loss of a secretion system (Zheng et al., 2008). The antigenic density between the two *M. b*ovis BCG strains was very similar (AAR = ~39) but it was higher than the antigenic density of the H37Rv (AAR = 40.6). In this case, the low virulence observed for the BCG strains is mainly attributed to the loss of RD1 locus, affecting the protein secretion pathway and the loss of cytolytic activity mediated by secreted ESAT-6, leading to reduced tissue invasiveness (Millington et al., 2011). However, the tendency to more antigenic density in the secretomes of BCG as compared to the H37Rv suggests why BCG strain has been the only one used as a vaccine so far. The higher antigenic density of all analyzed secretomes was the observed for the isolates 46 and 48 (AAR = ~37.5). This high antigenic density could be associated to the strong and sustained antigen stimulation for the granuloma formation and promoting the cell necrosis observed for Beijing strains (Flynn, 2004). Recently, it was suggested that *M. tuberculosis* uses mechanisms other than antigenic variation to evade T cells, indicating that antigenic variation is not a major mechanism of immune evasion in this pathogen (Coscolla et al., 2015). Interestingly, our data suggest that increasing the antigenic density of their secretomes could be one of the mechanisms associated with hypervirulent phenotypes of Beijing strains. In this regard, our survival assays performed in the tuberculosis murine model showed that isolates 46 and 48 have significantly lower survival rate than the one reported for the H37Rv (Hernandez-Pando et al., 1996). However, the antigenic density between two clinical isolates was very similar, AAR = 37.52 for isolate 46 and AAR = 37.55 for isolate 48, suggesting that the antigenic density is not the only mechanism responsible of the hypervirulent phenotype of these two Beijing strains.

The clinical isolates 46 and 48 showed a resistance to streptomycin and isoniazid, which are two of the first-line antibiotics used as treatment against Tuberculosis. It is necessary to explore not only the drugs acting at the intracellular level in *M. tuberculosis*, but also the drugs acting on proteins associated to the host-pathogen interactions. In this regard, it has been reported that several *M. tuberculosis* ES proteins interact with host cellular proteins to establish a successful infection modulating the host immune responses (Sreejit et al., 2014). The identification of this type of ES proteins may help us to design drugs against the host-pathogen interactions. For example, some virulence blockers are used to inhibit the secreted toxins of pathogens such as *Bacillus anthracis*, and *Clostridium tetani* (Moayeri et al., 2006; Clatworthy et al., 2007). Similarly, some studies have examined the potential of inhibiting extracellular molecules that participate in quorum sensing and help microorganisms such *Pseudomonas aeruginosa* in the biofilms formation (Duncan et al., 2012). Thus, the inhibition of ES proteins that are important for successful *M. tuberculosis* infection via disruption of host-pathogen interactions could help us to establish new opportunities for treatments against Tuberculosis.

In the core secretome we found homologous to known drug targets (Table S5), opening the possibility that known drugs could be used against ES proteins of *M. tuberculosis*. The list of drug targets includes the Ribonucleoside Reductase a homolog target used in cancer chemotherapy and several drugs including gallium nitrate and imexon were found to target this enzyme (Table S5). The gallium nitrate inhibits the activity of the Ribonucleoside Reductase and this drug has proven a high efficacy to treat Tuberculosis in murine models (Olakanmi et al., 2013). While imexon increases oxidative stress in target cells but also inhibits the Ribonucleoside Reductase (Roman et al., 2011). Nonetheless, there are not studies using this drug to Tuberculosis treatment. In basis on the above mentioned results, we suggest that several drugs (Table S5) usually used for cancer therapy, such as imexon and motexafin gadolinium could be explored as potential novel treatments against Tuberculosis through the modulation of the Ribonucleoside Reductase enzyme activity. However, it is important to mention that the human Ribonucleotide Reductase is also target of these cancer therapy drugs and secondary effects of a treatment using these drugs should be also evaluated. We also found several drugs that are in experimental phase (Table S5). Hence, their pharmacological action on the target protein is unknown. Some of them are: S-Oxy Cysteine, vitamin A, Pegvisomant, and Sofalcone which are target of the thiol peroxidase, short-chain dehydrogenase, cytochrome P450 (CYP139) and short chain dehydrogenase, respectively. Sofalcone suppress the production of NO and TNF in macrophages *in vitro* (Tanaka et al., 2009). Interestingly, these two citokines are responsible for the chronic inflammation and pneumonia in the late stages of *M. tuberculosis* infection. The CYP139 is immediately downstream of three polyketide synthase genes (pks17, 9, and 11) and upstream of genes encoding an ATP-binding ABC transporter, which is likely involved in the carriage (probably export) of macrolide molecules across the membrane (McLean and Munro, 2008). Hence, the CYP139 could be involved in the modification of these polyketide molecules prior to the transport process and it could be associated with the host-pathogen interactions.

The Ag85C is an essential protein for the cell wall synthesis in *M. tuberculosis* (Gobec et al., 2004). Recently, it was reported that Ebselen inhibits the growth of drug-resistant strains inhibiting the Ag85 complex (Favrot et al., 2013), demonstrating that it is an important target for the development of novel anti-Tuberculosis agents (Gobec et al., 2004). Thus, the two drugs reported in our analysis that also target the Ag85C (Table S5) could also be used to explore their effect on *M. tuberculosis*. Additionally, we also found a Class A beta-lactamase, which is a potential target for the Avibactam drug. Interestingly, it has been recently reported that clinical isolates of *M. tuberculosis* are susceptible to β-lactam/β–lactamase inhibitor combinations (Cohen et al., 2016). However, to date only the β-Lactamase inhibition by Avibactam has been proved in *M. abscessus* (Dubée et al., 2015). The Avibactam has no useful intrinsic antibacterial activity *per se*, it shows good results when it is combined with a β-lactam antibacterial as ceftazidime such as in the Avycaz which was recently used for treatment of intra-abdominal and urinary tract infections, including acute pyelonephritis, and it also has been used against carbapenemase-producing Enterobacteriaceae (Lucasti et al., 2013). These studies suggest that Avibactam in combination with other β-lactam antibiotics, such as Avycaz could be used against Tuberculosis.

The combination of SignalP, SecretomeP, TatP, and LipoP to predict the secreted proteins gives us the advantage to obtain proteins that were secreted through different secretion mechanisms and the use of TMHMM allows the elimination of secreted proteins containing transmembrane regions. We suggest that the combination of bioinformatics tools we designed allowed us a good match between experimental and predicted secretomes (Figure S2). For example, the secretome reported by Vizcaíno et al. (2010) also used the same 4 predictors that we used; however, they do not eliminate the transmembrane proteins in their secretome. While the secretome reported by Roy et al. (2013), only utilized the secreted proteins by SignalP followed by TATFIND1.4, PRED-LIPO, and the application of TMHMM analysis but only in a selected subgroup of proteins. Our bioinformatics pipeline could be useful to predict secretomes in other pathogen bacteria genomes. Undoubtedly, it would be ideal to support the secretome analysis with RNAseq data allowing us the identification of ES proteins that are differentially expressed during *M. tuberculosis* infection. For example, only the 41% of the *Taenia solium* secretome was reported as expressed (Gomez et al., 2015) while the 91% of the *E. multilocularis* secretome was differentially expressed between the parasite life-cycle stages (Wang et al., 2015). Our study contributes to increase the knowledge of the molecular mechanisms of host-pathogen interactions and we demonstrated how the ES proteins could be novel therapeutic targets against Tuberculosis using known drugs. Finally, a web server to calculate the AAR from protein datasets is under construction.

## Author contributions

Conceived and designed the experiments: FC, VC, AM, AO. Performed the experiments: FC, ZZ, VC, AM, CM, FS, AO. Analyzed the data: FC, VC, AO. Contributed reagents/materials/analysis tools: CM, LD, RH, AO. Wrote the paper: FC, ZZ, VC, AM, CM, FS, LD, RH, AO.

## Funding

We acknowledge the support provided by CONACyT grants CB- 2013-223279 and SALUD-2014-C01-234188 and CONACyT contract Fon.Inst./58/2016.

### Conflict of interest statement

The authors declare that the research was conducted in the absence of any commercial or financial relationships that could be construed as a potential conflict of interest.
