# Supplementary material for: Secretome Prediction of Two M. tuberculosis Clinical Isolates Reveals Their High Antigenic Density and Potential Drug Targets
Source: Front Microbiol. 2017 Feb 7;8:128. doi: 10.3389/fmicb.2017.00128 (PMC5293778; doi:10.3389/fmicb.2017.00128)
Supplement: Supplementary file 1 [file Table1.PDF]

S1 Table Statistic parameters for the best assemblies of isolates 46 and 48.

	Isolate 46	Isolate 48
# contigs	151	144
total length	4 294 183	4 302 876
N50	94 481	100 237
N75	56 108	62 123
NG50	92 019	100 237
NG75	55 775	59 430
# misassemblies	35	2369
# N's per 100 kbp	343.02	148.23
