# Supplementary material for: Secretome Prediction of Two M. tuberculosis Clinical Isolates Reveals Their High Antigenic Density and Potential Drug Targets
Source: Front Microbiol. 2017 Feb 7;8:128. doi: 10.3389/fmicb.2017.00128 (PMC5293778; doi:10.3389/fmicb.2017.00128)
Supplement: Supplementary file 2 [file Table2.PDF]

**S2 Table Top 5 most represented terms assigned by BLASTP for isolates 46, 48 and H37Rv strain of *M. tuberculosis* secretome**

| Term | # Isolate 46 proteins | # Isolate 48 proteins | # H37Rv proteins |
|---|---|---|---|
| Member of PPE family | 48 | 47 | 41 |
| Members of PE family | 16 | 16 | 13 |
| Members of PE-PGRS family | 16 | 26 | 29 |
| Lipoproteins | 32 | 35 | 22 |
| Oxidoreductases | 10 | 10 | 6 |
