# Supplementary material for: Secretome Prediction of Two M. tuberculosis Clinical Isolates Reveals Their High Antigenic Density and Potential Drug Targets
Source: Front Microbiol. 2017 Feb 7;8:128. doi: 10.3389/fmicb.2017.00128 (PMC5293778; doi:10.3389/fmicb.2017.00128)
Supplement: Supplementary file 3 [file Table3.PDF]

**S3A Table KEGG pathways assigned for isolate 46 ES proteins.**

| Ranking | Pathway name | Number of represented ES proteins (%) |
|---|---|---|
| 1 | ABC transporters | 7 (1.27) |
| 2 | Pyrimidine metabolism | 6 (1.08) |
| 3 | Ribosome | 6 (1.08) |
| 4 | Biosynthesis of amino acids | 5 (0.9) |
| 5 | Tuberculosis | 5 (0.9) |
| 6 | Amino sugar and nucleotide sugar metabolism | 4 (0.72) |
| 7 | Purine metabolism | 4 (0.72) |
| 8 | Starch and sucrose metabolism | 4 (0.72) |
| 9 | Aminobenzoate degradation | 3 (0.54) |
| 10 | Glyoxylate and dicarboxylate metabolism | 3 (0.54) |
| 11 | Mismatch repair | 3 (0.54) |
| 12 | Alanine, aspartate and glutamate metabolism | 2 (0.36) |
| 13 | Arginine and proline metabolism | 2 (0.36) |
| 14 | beta-Lactam resistance | 2 (0.36) |
| 15 | Biosynthesis of siderophore group non-ribosomal peptides | 2 (0.36) |
| 16 | Biotin metabolism | 2 (0.36) |
| 17 | Carbon metabolism | 2 (0.36) |
| 18 | Cationic antimicrobial peptide (CAMP) resistance | 2 (0.36) |
| 19 | Cysteine and methionine metabolism | 2 (0.36) |
| 20 | DNA replication | 2 (0.36) |
| 21 | Glycerophospholipid metabolism | 2 (0.36) |
| 22 | Glycine, serine and threonine metabolism | 2 (0.36) |
| 23 | Homologous recombination | 2 (0.36) |
| 24 | Nitrogen metabolism | 2 (0.36) |
| 25 | Penicillin and cephalosporin biosynthesis | 2 (0.36) |
| 26 | Peptidoglycan biosynthesis | 2 (0.36) |
| 27 | Peroxisome | 2 (0.36) |
| 28 | Sulfur metabolism | 2 (0.36) |
| 29 | Two-component system | 2 (0.36) |
| 30 | Valine, leucine and isoleucine degradation | 2 (0.36) |
| 31 | Amyotrophic lateral sclerosis (ALS) | 1 (0.18) |
| 32 | Arginine biosynthesis | 1 (0.18) |
| 33 | Biosynthesis of unsaturated fatty acids | 1 (0.18) |
| 34 | Biosynthesis of vancomycin group antibiotics | 1 (0.18) |
| 35 | Bisphenol degradation | 1 (0.18) |
| 36 | Carbon fixation pathways in prokaryotes | 1 (0.18) |
| 37 | Cyanoamino acid metabolism | 1 (0.18) |
| 38 | D-Arginine and D-ornithine metabolism | 1 (0.18) |
| 39 | Ether lipid metabolism | 1 (0.18) |
| 40 | Fatty acid biosynthesis | 1 (0.18) |

| | | |
|---|---|---|
| 41 | Fatty acid degradation | 1 (0.18) |
| 42 | Fatty acid metabolism | 1 (0.18) |
| 43 | Fructose and mannose metabolism | 1 (0.18) |
| 44 | GABAergic synapse | 1 (0.18) |
| 45 | Glucagon signaling pathway | 1 (0.18) |
| 46 | Glutamatergic synapse | 1 (0.18) |
| 47 | Glutathione metabolism | 1 (0.18) |
| 48 | Glycosaminoglycan degradation | 1 (0.18) |
| 49 | Histidine metabolism | 1 (0.18) |
| 50 | Huntington's disease | 1 (0.18) |
| 51 | Inositol phosphate metabolism | 1 (0.18) |
| 52 | Insulin resistance | 1 (0.18) |
| 53 | Insulin signaling pathway | 1 (0.18) |
| 54 | Limonene and pinene degradation | 1 (0.18) |
| 55 | Longevity regulating pathway - multiple species | 1 (0.18) |
| 56 | Lysine biosynthesis | 1 (0.18) |
| 57 | Monobactam biosynthesis | 1 (0.18) |
| 58 | Nucleotide excision repair | 1 (0.18) |
| 59 | Phenylalanine metabolism | 1 (0.18) |
| 60 | Phenylalanine, tyrosine and tryptophan biosynthesis | 1 (0.18) |
| 61 | Polycyclic aromatic hydrocarbon degradation | 1 (0.18) |
| 62 | Polyketide sugar unit biosynthesis | 1 (0.18) |
| 63 | Prion diseases | 1 (0.18) |
| 64 | Propanoate metabolism | 1 (0.18) |
| 65 | Riboflavin metabolism | 1 (0.18) |
| 66 | RNA polymerase | 1 (0.18) |
| 67 | Selenocompound metabolism | 1 (0.18) |
| 68 | Stilbenoid, diarylheptanoid and gingerol biosynthesis | 1 (0.18) |
| 69 | Streptomycin biosynthesis | 1 (0.18) |
| 70 | Styrene degradation | 1 (0.18) |
| 71 | Sulfur relay system | 1 (0.18) |
| 72 | Taurine and hypotaurine metabolism | 1 (0.18) |
| 73 | Thyroid hormone signaling pathway | 1 (0.18) |
| 74 | Tryptophan metabolism | 1 (0.18) |

**S3B Table KEGG pathways assigned for isolate 48 ES proteins.**

| Ranking | Pathway name | Number of represented ES proteins (%) |
|---|---|---|
| 1 | ABC transporters | 5 (0.96) |
| 2 | Pyrimidine metabolism | 5 (0.96) |
| 3 | Ribosome | 5 (0.96) |
| 4 | Tuberculosis | 5 (0.96) |
| 5 | Amino sugar and nucleotide sugar metabolism | 4 (0.77) |

| | | |
|---|---|---|
| 6 | Glycine, serine and threonine metabolism | 4 (0.77) |
| 7 | Biosynthesis of amino acids | 3 (0.58) |
| 8 | Glyoxylate and dicarboxylate metabolism | 3 (0.58) |
| 9 | Peptidoglycan biosynthesis | 3 (0.58) |
| 10 | Alanine, aspartate and glutamate metabolism | 2 (0.39) |
| 11 | Aminobenzoate degradation | 2 (0.39) |
| 12 | Arginine and proline metabolism | 2 (0.39) |
| 13 | beta-Lactam resistance | 2 (0.39) |
| 14 | Biosynthesis of siderophore group nonribosomal peptides | 2 (0.39) |
| 15 | Carbon metabolism | 2 (0.39) |
| 16 | Cationic antimicrobial peptide (CAMP) resistance | 2 (0.39) |
| 17 | Glycerophospholipid metabolism | 2 (0.39) |
| 18 | Nitrogen metabolism | 2 (0.39) |
| 19 | Penicillin and cephalosporin biosynthesis | 2 (0.39) |
| 20 | Peroxisome | 2 (0.39) |
| 21 | Purine metabolism | 2 (0.39) |
| 22 | Two-component system | 2 (0.39) |
| 23 | Valine, leucine and isoleucine degradation | 2 (0.39) |
| 24 | Alcoholism | 1 (0.19) |
| 25 | Amphetamine addiction | 1 (0.19) |
| 26 | Amyotrophic lateral sclerosis (ALS) | 1 (0.19) |
| 27 | Arginine biosynthesis | 1 (0.19) |
| 28 | Biosynthesis of unsaturated fatty acids | 1 (0.19) |
| 29 | Biosynthesis of vancomycin group antibiotics | 1 (0.19) |
| 30 | Biotin metabolism | 1 (0.19) |
| 31 | Bisphenol degradation | 1 (0.19) |
| 32 | Carbon fixation in photosynthetic organisms | 1 (0.19) |
| 33 | Carbon fixation pathways in prokaryotes | 1 (0.19) |
| 34 | Cocaine addiction | 1 (0.19) |
| 35 | Cyanoamino acid metabolism | 1 (0.19) |
| 36 | Cysteine and methionine metabolism | 1 (0.19) |
| 37 | D-Arginine and D-ornithine metabolism | 1 (0.19) |
| 38 | Dopaminergic synapse | 1 (0.19) |
| 39 | Drug metabolism - cytochrome P450 | 1 (0.19) |
| 40 | Ether lipid metabolism | 1 (0.19) |
| 41 | Fatty acid biosynthesis | 1 (0.19) |
| 42 | Fatty acid metabolism | 1 (0.19) |
| 43 | Fructose and mannose metabolism | 1 (0.19) |
| 44 | GABAergic synapse | 1 (0.19) |
| 45 | Glutamatergic synapse | 1 (0.19) |
| 46 | Glutathione metabolism | 1 (0.19) |
| 47 | Glycosaminoglycan degradation | 1 (0.19) |
| 48 | Histidine metabolism | 1 (0.19) |
| 49 | Huntington's disease | 1 (0.19) |
| 50 | Inositol phosphate metabolism | 1 (0.19) |
| 51 | Isoquinoline alkaloid biosynthesis | 1 (0.19) |

| | | |
|---|---|---|
| 52 | Limonene and pinene degradation | 1 (0.19) |
| 53 | Longevity regulating pathway - multiple species | 1 (0.19) |
| 54 | Mismatch repair | 1 (0.19) |
| 55 | Nucleotide excision repair | 1 (0.19) |
| 56 | Pentose and glucuronate interconversions | 1 (0.19) |
| 57 | Pentose phosphate pathway | 1 (0.19) |
| 58 | Phenylalanine metabolism | 1 (0.19) |
| 59 | Polycyclic aromatic hydrocarbon degradation | 1 (0.19) |
| 60 | Polyketide sugar unit biosynthesis | 1 (0.19) |
| 61 | Porphyrin and chlorophyll metabolism | 1 (0.19) |
| 62 | Prion diseases | 1 (0.19) |
| 63 | Propanoate metabolism | 1 (0.19) |
| 64 | RNA polymerase | 1 (0.19) |
| 65 | Selenocompound metabolism | 1 (0.19) |
| 66 | Serotonergic synapse | 1 (0.19) |
| 67 | Starch and sucrose metabolism | 1 (0.19) |
| 68 | Stilbenoid, diarylheptanoid and gingerol biosynthesis | 1 (0.19) |
| 69 | Streptomycin biosynthesis | 1 (0.19) |
| 70 | Sulfur metabolism | 1 (0.19) |
| 71 | Sulfur relay system | 1 (0.19) |
| 72 | Taurine and hypotaurine metabolism | 1 (0.19) |
| 73 | Thyroid hormone signaling pathway | 1 (0.19) |
| 74 | Tryptophan metabolism | 1 (0.19) |
| 75 | Tyrosine metabolism | 1 (0.19) |

**S3C Table KEGG pathways assigned for H37Rv ES proteins.**

| Ranking | Pathway name | Number of represented ES proteins (%) |
|---|---|---|
| 1 | ABC transporters | 8 (1.46) |
| 2 | Ribosome | 6 (1.09) |
| 3 | Pyrimidine metabolism | 5 (0.91) |
| 4 | Tuberculosis | 5 (0.91) |
| 5 | Amino sugar and nucleotide sugar metabolism | 4 (0.73) |
| 6 | Glycine, serine and threonine metabolism | 4 (0.73) |
| 7 | Biosynthesis of amino acids | 3 (0.55) |
| 8 | Glyoxylate and dicarboxylate metabolism | 3 (0.55) |
| 9 | Carbon metabolism | 2 (0.36) |
| 10 | Nitrogen metabolism | 2 (0.36) |
| 11 | Sulfur metabolism | 2 (0.36) |
| 12 | Glycerophospholipid metabolism | 2 (0.36) |
| 13 | Purine metabolism | 2 (0.36) |
| 14 | Alanine, aspartate and glutamate metabolism | 2 (0.36) |
| 15 | Valine, leucine and isoleucine degradation | 2 (0.36) |
| 16 | Arginine and proline metabolism | 2 (0.36) |

| | | |
|---|---|---|
| 17 | Peptidoglycan biosynthesis | 2 (0.36) |
| 18 | Biosynthesis of siderophore group nonribosomal peptides | 2 (0.36) |
| 19 | Penicillin and cephalosporin biosynthesis | 2 (0.36) |
| 20 | Mismatch repair | 2 (0.36) |
| 21 | Two-component system | 2 (0.36) |
| 22 | Peroxisome | 2 (0.36) |
| 23 | beta-Lactam resistance | 2 (0.36) |
| 24 | Fatty acid metabolism | 1 (0.18) |
| 25 | Pentose phosphate pathway | 1 (0.18) |
| 26 | Pentose and glucuronate interconversions | 1 (0.18) |
| 27 | Fructose and mannose metabolism | 1 (0.18) |
| 28 | Starch and sucrose metabolism | 1 (0.18) |
| 29 | Propanoate metabolism | 1 (0.18) |
| 30 | Inositol phosphate metabolism | 1 (0.18) |
| 31 | Carbon fixation in photosynthetic organisms | 1 (0.18) |
| 32 | Carbon fixation pathways in prokaryotes | 1 (0.18) |
| 33 | Fatty acid biosynthesis | 1 (0.18) |
| 34 | Ether lipid metabolism | 1 (0.18) |
| 35 | Biosynthesis of unsaturated fatty acids | 1 (0.18) |
| 36 | Cysteine and methionine metabolism | 1 (0.18) |
| 37 | Arginine biosynthesis | 1 (0.18) |
| 38 | Histidine metabolism | 1 (0.18) |
| 39 | Tyrosine metabolism | 1 (0.18) |
| 40 | Phenylalanine metabolism | 1 (0.18) |
| 41 | Tryptophan metabolism | 1 (0.18) |
| 42 | Taurine and hypotaurine metabolism | 1 (0.18) |
| 43 | Selenocompound metabolism | 1 (0.18) |
| 44 | Cyanoamino acid metabolism | 1 (0.18) |
| 45 | D-Arginine and D-ornithine metabolism | 1 (0.18) |
| 46 | Glutathione metabolism | 1 (0.18) |
| 47 | Glycosaminoglycan degradation | 1 (0.18) |
| 48 | Riboflavin metabolism | 1 (0.18) |
| 49 | Biotin metabolism | 1 (0.18) |
| 50 | Porphyrin and chlorophyll metabolism | 1 (0.18) |
| 51 | Polyketide sugar unit biosynthesis | 1 (0.18) |
| 52 | Biosynthesis of vancomycin group antibiotics | 1 (0.18) |
| 53 | Isoquinoline alkaloid biosynthesis | 1 (0.18) |
| 54 | Streptomycin biosynthesis | 1 (0.18) |
| 55 | Aminobenzoate degradation | 1 (0.18) |
| 56 | Drug metabolism - cytochrome P450 | 1 (0.18) |
| 57 | RNA polymerase | 1 (0.18) |
| 58 | Sulfur relay system | 1 (0.18) |
| 59 | DNA replication | 1 (0.18) |
| 60 | Nucleotide excision repair | 1 (0.18) |
| 61 | Homologous recombination | 1 (0.18) |
| 62 | Thyroid hormone signaling pathway | 1 (0.18) |

| | | |
|---|---|---|
| 63 | Glutamatergic synapse | 1 (0.18) |
| 64 | GABAergic synapse | 1 (0.18) |
| 65 | Dopaminergic synapse | 1 (0.18) |
| 66 | Serotonergic synapse | 1 (0.18) |
| 67 | Longevity regulating pathway - multiple species | 1 (0.18) |
| 68 | Amyotrophic lateral sclerosis (ALS) | 1 (0.18) |
| 69 | Huntington's disease | 1 (0.18) |
| 70 | Prion diseases | 1 (0.18) |
| 71 | Cocaine addiction | 1 (0.18) |
| 72 | Amphetamine addiction | 1 (0.18) |
| 73 | Alcoholism | 1 (0.18) |
| 74 | Chagas disease (American trypanosomiasis) | 1 (0.18) |
| 75 | African trypanosomiasis | 1 (0.18) |
| 76 | Cationic antimicrobial peptide (CAMP) | 1 (0.18) |
