# Supplementary material for: Secretome Prediction of Two M. tuberculosis Clinical Isolates Reveals Their High Antigenic Density and Potential Drug Targets
Source: Front Microbiol. 2017 Feb 7;8:128. doi: 10.3389/fmicb.2017.00128 (PMC5293778; doi:10.3389/fmicb.2017.00128)
Supplement: Supplementary file 6 [file Image1.PDF]

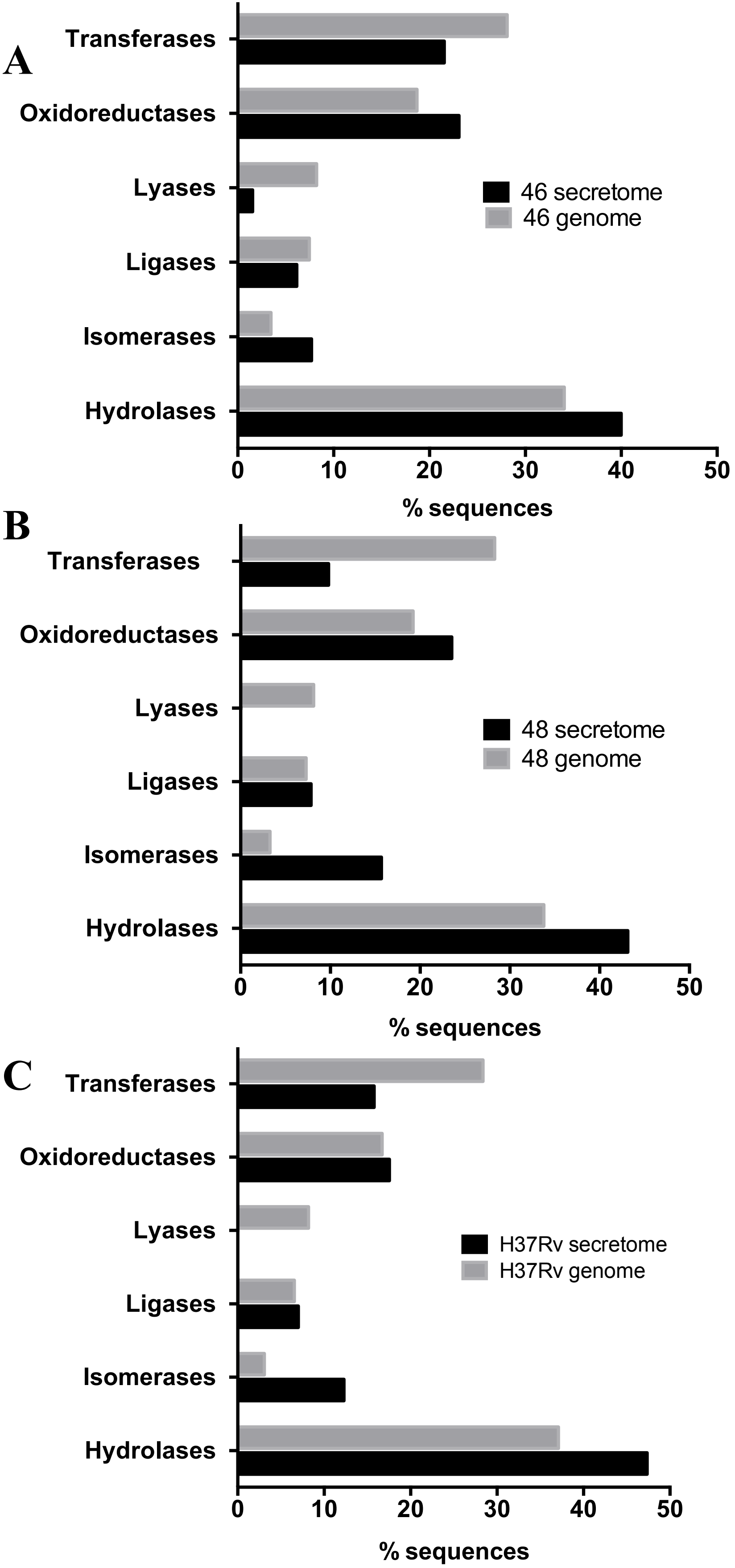

S1 Fig. Percentage of sequences classified in an Enzyme Commission (EC) class for secretome and genome proteins. Isolate 46 **(A)**, isolate 48 **(B)** and reference strain H37Rv **(C)** .
