# Supplementary material for: Secretome Prediction of Two M. tuberculosis Clinical Isolates Reveals Their High Antigenic Density and Potential Drug Targets
Source: Front Microbiol. 2017 Feb 7;8:128. doi: 10.3389/fmicb.2017.00128 (PMC5293778; doi:10.3389/fmicb.2017.00128)
Supplement: Supplementary file 7 [file Image2.PDF]

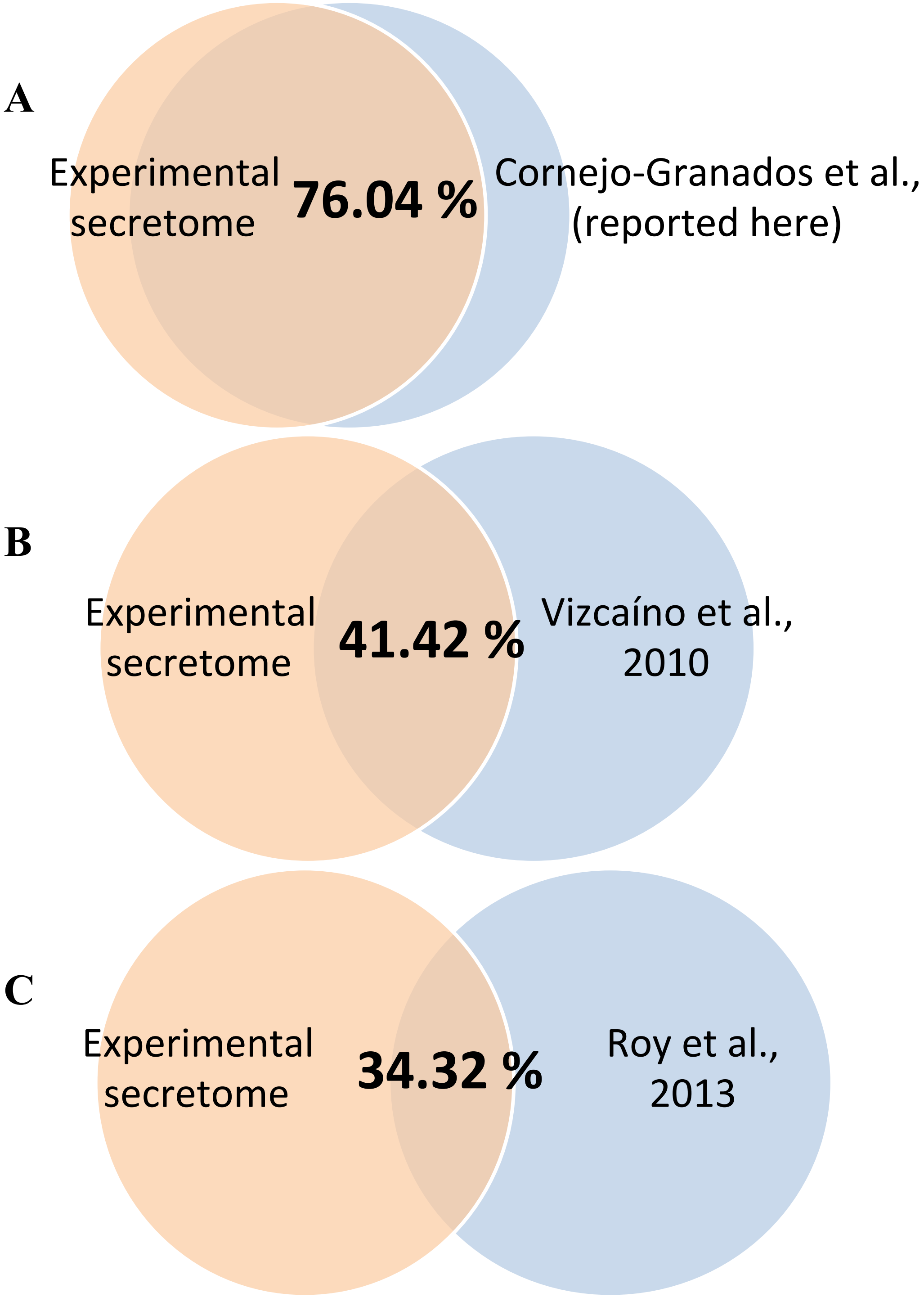


**S2 Fig. Venn diagram of the experimental secretome shared with predicted H37Rv secretomes.** The percentage of the experimental secretome composed of 338 proteins that is common between the different reported secretomes is shown in the intersection. This data was obtained by a BLASTP analysis (E-value $1.0E^{-3}$) of the 338 proteins against the different secretomes. **A)** The experimental secretome shares 257 proteins (76.04%) with the predicted secretome reported here. **B)** The experimental secretome shares 140 proteins (41.42%) with the secretome reported by Vizcaíno et al., 2010. **C)** The experimental secretome shares 116 proteins (34.32%) with the secretome reported by Roy et al., 2013.
